# Flywheel squats versus free weight high load squats for improving high velocity movements in football. A randomized controlled trial

**DOI:** 10.1186/s13102-020-00210-y

**Published:** 2020-10-02

**Authors:** Edvard H. Sagelv, Sigurd Pedersen, Lars Petter R. Nilsen, Andrea Casolo, Boye Welde, Morten B. Randers, Svein Arne Pettersen

**Affiliations:** 1 School of Sport Sciences, Faculty of Health Sciences, UiT the Arctic University of Norway, Tromsø, Norway; 2 Department of Movement, Human and Health Sciences, University of Rome ‘Foro Italico’, Rome, Italy; 3 Department of Bioengineering, Imperial College London, London, UK; 4 Department of Sport Sciences and Clinical Biomechanics, Faculty of Health Sciences, University of Southern Denmark, Odense, Denmark

**Keywords:** male1, soccer2, Maximal strength3, sprint4, jump5, power6

## Abstract

**Background:**

High load (HL: > 85% of one repetition maximum (1RM)) squats with maximal intended velocity contractions (MIVC) combined with football sessions can be considered a relevant and time-efficient practice for maintaining and improving high velocity movements in football. Flywheel (FW) resistance exercise (RE) have recently emerged with promising results on physical parameters associated with football performance.

**Methods:**

In this randomized controlled trial over 6 weeks, 38 recreationally active male football players randomly performed RE with MIVCs two times per week as either 1) FW squats (*n* = 13) or 2) barbell free weight (BFW) HL squats (*n* = 13), where a third group served as controls (*n* = 12). All three groups conducted 2–3 football sessions and one friendly match a week during the intervention period. Pre- to post changes in 10-m sprint, countermovement jump (CMJ) and 1RM partial squat were assessed with univariate analyses of variance.

**Results:**

The FW and BFW group equally improved their 10-m sprint time (2 and 2%, respectively, within group: both *p* < 0.001) and jump height (9 and 8%, respectively, within group: both *p* < 0.001), which was superior to the control group’s change (between groups: both *p* < 0.001). The BFW group experienced a larger increase (46%) in maximal squat strength than the FW group (17%, between groups: *p* < 0.001), which both were higher than the control group’s change (both *p* < 0.001).

**Conclusion:**

Squats carried out with FWs or BFWs where both are performed with MIVCs and combined with football sessions, were equally effective in improving sprint time and jump height in football players. The BFW group experienced a more than two-fold larger increase in maximal partial squat strength than the FW group in maximal partial squat strength. This presents FW RE as an alternative to BFW HL RE for improving high velocity movements in football.

**Trial registration:**

ClinicalTrials.gov Identifier: NCT04113031 (retrospectively registered, date: 02.10.2019).

## Background

Maximal and high velocity forces are considered decisive for human movement performance. In modern football, the importance of performing rapid and high velocity movements, such as sprints and jumps, has gradually increased [1–5]. Maximal lower limb muscle strength is associated with lower limb muscle power [6, 7], where an increase in lower limb muscle strength is likely to result in an increased sprint performance [8].

High power resistance exercise (RE) with high velocities and low external loads is effective for improving rapid and high velocity movements [8–11]. However, independent of external loads, the intention of maximal velocity while performing RE is likely the most prominent factor for increasing the neural drive to the muscles, resulting in an increased velocity in the mechanical response [8, 12–14]. This is likely explained by short time to peak tensions, high rates of torque development, high motor unit discharge rates and an early and fast motor unit recruitment [15–18]. Consequently, RE with high external loads (HL: ≥85% of 1RM) and subsequently low velocity movement is also likely effective, as long as the intended velocity during the contractions is maximal [13]. In fact, HL RE is reported to be effective in football players when it is combined with performing high velocity movements (e.g. sprints and jumps) in football practice [8, 19]. Additionally, although the intensity in HL RE is high, the low number of repetitions and sets allows the total RE volume to be low. Due to the challenges of incorporating all important physical aspects while also ensuring sufficient recovery time in football players’ weekly exercise and competitive schedules [20], HL RE can be considered a relevant and time-efficient exercise modality for maintaining [21] and improving [22, 23] sprint and jump performance in football.

As eccentric muscle contractions allows for higher force production compared to the concentric contractions [24, 25], exercises with eccentric overload, such as inertia spinning YoYo™ flywheel (FW) devices [26], have been suggested as an alternative or supplement to the established exercise modalities [24, 25]. In FW devices, a band is connected to a pivoting shaft, where pulling the band unwinds the band and kinetic energy is subsequently produced in the shaft due to the inertia of the spinning FW. When the band reaches its maximal length, the FW keeps spinning and rewinds the band again and high muscle force is produced during the eccentric phase if the individual is trying to slow the spinning of the FW, where peak muscles forces are produced if the individual is instructed to break the eccentric movement towards the end of the rewound band [26, 27].

Over the past two decades, a substantial number of studies have assessed the utility of FW RE for improving sports performance, with positive effects on maximal strength, muscle power, jump height, sprint performance and changes of direction movements [26, 27]. Although the evidence for improved performance is compelling, there are fewer studies comparing FW RE to other RE modalities, which is necessary to determine whether FW exercise could have similar effects compared with the established RE modalities.

To our knowledge, no study has compared the effect of FW RE versus free weight RE using the same motion path, which consequently stimulates the same muscles. Additionally, no study has compared the effect of FW exercise and free weight using HL with maximal intended velocity contraction (MIVC)s combined with football sessions, which can be considered a relevant and time-efficient exercise modality for improving high velocity movements in football while also improving maximal strength [22, 23]. Such information can be highly applicable for coaches in football clubs, who should use the best practice in relation to total exercise load to optimize performance of the players, at least in elite clubs. Thus, the objective of this study was to compare the effect of FW RE versus free weight HL RE on 10-m sprint time, countermovement jump (CMJ), and 1RM partial 90° range of motion (ROM) squat strength in football players. In this randomized controlled trial, both the FW RE and the free weight RE were carried out in a squat exercise with MIVCs and combined with football sessions. We hypothesized 1) that RE using FW and barbell free weight (BFW) combined with football practices will equally improve sprint time and jump height, and 2) that squats carried out in a BFW exercise will result in superior improvements in 1RM partial squat compared with FW squats.

## Methods

### Design

In this randomized controlled trial, we randomly allocated 49 players into three different groups using Research Randomizer [28] (three sets, 17 numbers per set, ID-number range 1–49, *“every number unique”*, *“no sorted order”* and *“no place marker”*); 1) flywheel (FW) group (*n* = 16), 2) barbell free weight (BFW) group (*n* = 16) and 3) control group (*n* = 17). Due to drop out (22.5%), the final number in the three groups were 13, 13 and 12 players in the FW, BFW and control group, respectively. The FW and the BFW group participated in an intervention where they performed a squat exercise either with a FW device or with BFWs twice a week over 6 weeks (in total 12 sessions) as part of their preseason preparations. The control group was instructed not to perform lower body RE and only to perform their teams’ preseason preparations and acted as controls. During the intervention period, all enrolled players were instructed to avoid complementary REs for their lower body, while no restrictions were given regarding REs for their upper body. Our outcome measures were 10-m sprint time, CMJ and 1RM partial squat, which we measured pre- and post the 6 week long intervention.

This study was carried out in accordance to the Declaration of Helsinki; prior to pre-tests, all the players were informed of the purpose of the study and its associated risks and benefits, before providing oral and written informed consent. The Norwegian Data Protection Service approved the study and the storage of personal data (Approval reference number: 374030), without further Regional Ethical approval per applicable institutional and national guidelines for sport and exercise science [29, 30].

### Subjects

**Fig. 1:**
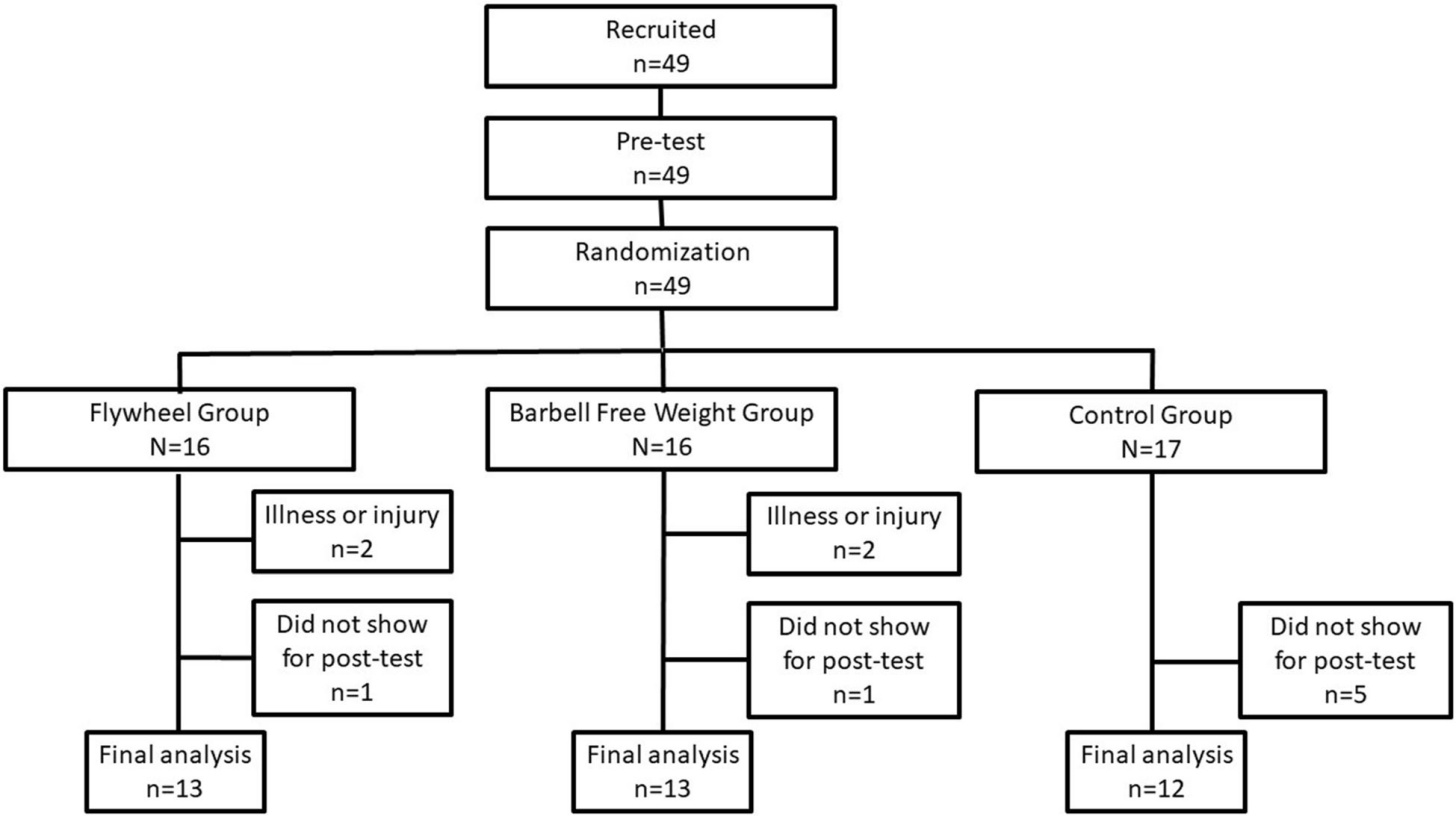
The flow and random allocation of participants

In the pre-season period in Norway, from January to March 2019, 49 recreationally active football players volunteered to participate. Recruitment period was January 5th to January 31st, data collection was from February 1st to March 31st. The players played at the two highest regional levels in the Norwegian national league system, which is the 5th and 6th levels in Norway. After contacting multiple 5th and 6th level teams’ coaches, the included players were recruited from teams with similar overall exercise load with the following inclusion criteria; 1) two or three 60 min football sessions and 2) one friendly football match a week. Exclusion criteria was no injury or disease preventing from participation in RE and football practice. The flow and random allocation of participants are illustrated in Fig. 1. Four of the 49 recruited players reported to be unfamiliar with RE, while the remaining players reported to perform 1–6 weekly RE sessions beside their teams’ football sessions. Four players withdrew from the study prior to study completion due to illness and injuries not related to the study interventions, and seven players did not show up for post-tests. As a result, 38 players completed the study. The descriptive baseline test characteristics are shown in Table 1; there were no differences in baseline characteristics between the intervention groups (all *p* ≥ 0.20).

**Table 1:** Baseline descriptive characteristics of the football players expressed by group

	FW group (***n*** = 13)	BFW group (***n*** = 13)	Control group (***n*** = 12)
Age (yr)	23.07 ± 3.15	23.23 ± 2.12	25.3 ± 2.39
Body mass (kg)	78.69 ± 7.42	78.87 ± 11.98	83.13 ± 7.06
Height (m)	1.81 ± 0.05	1.81 ± 0.05	1.82 ± 0.04
BMI (kg/m^2^)	24.08 ± 1.85	24.22 ± 2.44	25.21 ± 1.70
RE volume per week (hr)	2.3 ± 1.50	3.0 ± 2.13	2.3 ± 1.78
Playing Level			
Fifth level (n)	12	13	10
Sixth level (n)	4	3	2
Playing position			
Goal Keeper (n)	2	1	N/A
Central back (n)	4	3	3
Full back (n)	3	2	4
Central midfielder (n)	4	4	5
Wide midfielder (n)	3	3	2
Striker (n)	1	3	2

Data are shown as mean ± SD. *FW* Flywheel, *BFW* Barbell free weight, *RE* Resistance exercise, *BMI* Body mass index

### Test procedures

Prior to the interventions, the players underwent pre-tests in the following order on the same test day: 1) 10-m sprint time, 2) CMJ and 3) 1RM in a barbell free weight partial squat, carried out as 90° ROM in the knee joint (standing position = 180°). The players’ height was assessed on a portable scale (Seca 217, Seca GmbH & Co., KG, Germany) and body mass on a portable force platform (Hurlab FP4, HUR Labs Oy, Kokkola, Finland), which was connected to a portable laptop (ThinkPad, Lenovo Group Ltd., Beijing, China) through a USB cable and monitored with the manufacturer’s software (Force platform software suite, HUR Labs Oy, Kokkola, Finland). Body mass index (BMI) was calculated. Prior to testing, the players jogged for 15 min at progressively increasing intensity (easy to moderately paced jogging) with various exercises (e.g. knee raises, heel kicks, lunges, and frontal vertical kicks to their hands) on artificial grass, supervised by an instructor. The subjects wore jogging shoes and light clothing. Following the 15 min jog, the players performed two progressive 15-m sprints instructed to be at 95% of self-determined maximum acceleration.

### 10-m sprint test

The 10-m sprint test was performed on artificial grass indoors. Single-beam photocells (ATU-X, IC Control AB, Stockholm, Sweden), mounted to the floor and walls recorded the sprint times, where the photocells at the starting and the finishing line were placed 20 cm and 100 cm above the ground, respectively. Within-subject coefficient of variation of single-beam photocells is reported to be 2% [31]. A marker was placed 30 cm behind the starting timing gate, where the players chose their starting position behind the marker. The players started the test on their own initiative, and without verbal encouragement, by breaking the laser beam at the starting timing gate and sprinted to the finishing line as fast as they could. Each player was given three attempts with 3 min recovery between each sprint. The fastest sprint time was recorded.

### Countermovement jump

Following ≥3 min rest from the sprint test, the players performed the CMJ test on the portable force platform (Hurlab FP4, HUR Labs Oy, Kokkola, Finland) following the body mass measurement. Portable force platforms is found to measure CMJ jump height within a 2% accuracy compared to a laboratory floor mounted force platform (Type 9281B Kistler, Instrumente AG, Winterthur, Switzerland) [32] and with a 2.8% within-subject coefficient of variation [33]. Starting from an upright standing position with their feet shoulder-width apart and with both hands placed on their hips, the players were instructed to make a preliminary downward movement (eccentric phase) by flexing their knees to approximately 90° (knee-flexion) before performing the concentric phase of the vertical jump off the ground by extending the knees and the hips, respectively. Each player was given three attempts with 3 min recovery between each jump. If an incorrect jump was performed (e.g. typical mistake was lifting the heel prior to extending the knees), the player was given a new attempt. The force platform measures the vertical jump height in centimetres (cm) by calculating the centre of mass displacement from force development (take-off velocity) and body mass. The sampling rate was set to 1200 Hz. The highest vertical jump was recorded.

### One repetition maximum in partial squat

Following the CMJ test, the players performed a partial ROM (approximately 90° knee joint angle) back squat test using an Olympic barbell (Eleiko, Halmstad, Sweden) for the assessment of 1RM. We used a slightly modified 1RM protocol used by Helgerud et al. [34]. The players first warmed up by lifting the Olympic barbell (20 kg) without additional weights for 8–10 repetitions, and thereafter performing two sets of progressively decreasing repetitions (6 and 3 repetitions, respectively) and increasing the weights based on their perceived effort in the previous warm-up set (Helgerud et al. [34] specified no 1RM warm up). Thereafter, the players attempted their 1RM trials with increasing weights (10 kg) until failure (Helgerud et al. [34] used 5 kg increments). Failure was defined as inability to lift the barbell to standing (starting) position (180° knee joint angle). A mechanical goniometer was held to the lateral part of their knee joint by an instructor to ensure that the players reached 90° of knee flexion before they were given a verbal “go” and they could start the concentric phase of the lift. The kilograms (kg) lifted in the last approved lift with one repetition was considered their 1RM and recorded in kg. One repetition maximum was normally reached between 3 and 6 sets, ≥3 min recovery was given between each attempt. The coefficient of variation for 1RM squat is reported to be 2.9% [35]. As 1RM strength divided by body mass may be imprecise where a heavier individual may be overestimated and a lighter individual underestimated [34, 36], the kg lifted was also allometrically scaled as kg lifted in the squat exercise multiplied by body mass raised to the power of 0.67 (kg lifted·kg body mass^-0.67^) [34, 36].

### Exercise interventions

An overview of the exercise programs is presented in Table 2. Over the course of the interventions, all players in all three groups were instructed to adhere to their two-three weekly football sessions and friendly matches of their team. The players in the FW and BFW groups started their RE interventions the week following pre-tests, which did not coincide with their football sessions (i.e. RE and football sessions was separate). Prior to both intervention groups’ sessions, the players performed a 10 min self-selected low intensity aerobic warm-up on a motorized treadmill (ELG 70, Woodway Inc., Waukesha,Wisconsin, United States) or an ergometer bike (Pro/Trainer, Wattbike Ltd., Nottingham, United Kingdom). For both groups, the players were instructed to perform their exercise with MIVCs and were given verbal encouragement throughout the sessions. All intervention sessions were performed in the same laboratory and supervised by the same instructor. The players in both intervention groups were expected to experience a large increase in knee extensor strength. Therefore, the players performed the Nordic hamstring exercise to avoid a large quadriceps-to-hamstring strength ratio and thereby potentially reduce the risk for hamstring strains [37].

**Table 2:** The exercise programs of the interventions

	Flywheel group	Barbell Free Weight group	Control Group
Equipment	Flywheel Device	Olympic Barbell, free weight and squat rack.	N/A
**Exercise sets and repetitions (intensity)**			
*Week 1 (Familiarization sessions)*			
Intervention exercise	3 × 6 (inertia #1, #2, #3 or #4)	3 × 8 (~ 70% of 1RM)	N/A
Nordic Hamstring	3 × 4 (Body weight)	3 × 4 (Body weight)	N/A
*Week 2*			
*Criteria for increasing load*	*An average > 4 watts·kg*^*−1*^ *from each repetition of one set*	*If they could perform five repetitions within one set*	*N/A*
Intervention exercise	3 × 6 (inertia #1, #2, #3 or #4)	4 × 4 (> 85% of 1RM)	N/A
Nordic Hamstring	3 × 5	3 × 5 (Body weight)	N/A
*Week 3*			
Intervention exercise	3 × 5 (inertia #1, #2, #3 or #4)	4 × 4 (> 85% of 1RM)	N/A
Nordic Hamstring	3 × 6 (Body weight)	3 × 6 (Body weight)	N/A
*Week 4*			
Intervention exercise	4 × 5 (inertia #1, #2, #3 or #4)	4 × 4 (> 85% of 1RM)	N/A
Nordic Hamstring	3 × 6 (Body weight)	3 × 6 (Body weight)	N/A
*Week 5*			
Intervention exercise	4 × 4 (inertia #1, #2, #3 or #4)	4 × 4 (> 85% of 1RM)	N/A
Nordic Hamstring	3 × 8 (Body weight)	3 × 8 (Body weight)	N/A
*Week 6*			
Intervention exercise	4 × 4 (inertia #1, #2, #3 or #4)	4 × 4 (> 85% of 1RM)	N/A
Nordic Hamstring	3 × 10 (Body weight)	3 × 10 (Body weight)	N/A

*N/A* Not applicable. Inertia #1 = 0.025 kg·m^−2^, #2 = 0.05 kg·m^− 2^, #3 = 0.075 kg·m^− 2^ or #4 = 0.1 kg·m^− 2^

The Nordic hamstring exercise was performed at the end of each exercise session (for both interventions) and involved three sets of four repetitions (week 1) where the number of repetitions were progressively increased to five in week 2, six in week 3–4, eight in week 5, and 10 in the final week of the interventions. At the end of the 6-week exercise interventions, the participants performed post-tests in the same order as the pre-tests.

### Flywheel squat group

The players allocated to the FW group was equipped with a vest on their upper body connected with a band to the FW device (#215 YoYo Squat Unlimited Pro, nHance, YOYO Technology, Stockholm, Sweden). In the FW device, different sized spinning inertia FWs can be connected to the pivoting shaft (size #0.5: 0.0125 kg·m^− 2^, #1: 0.025 kg·m^− 2^, 2#: 0.05 kg·m^− 2^, 4#: 0.1 kg·m^− 2^). The first two sessions (week 1) were familiarization sessions, which consisted of three sets with six repetitions. Thereafter, from week 2, the players performed three sets with six repetitions with MIVCs followed by 3 × 5, 4 × 5 and 4 × 4 repetitions in week 3, 4 and 5–6, respectively. Recovery time between sets was set to ≥3 min. The players started in a partial squat position (~ 90° knee angle) and performed first a standardized warm-up set with six repetitions using the #1 inertia FW (0.025 kg·m^− 2^). In all exercise sets, the players started with three slow repetitions to get into the flow of the squat exercise movement before beginning their scheduled MIVC sets (6 × 3, 5 × 3, 4 × 5, 4 × 4 depending on exercise week), where they were given a verbal “go” when starting to push with MIVCs from starting position (~ 90° knee joint angle) to standing position. The band connecting the vest and the FW device was strapped tightly making the players stop at approximately 175° knee joint angle in the standing position when the FW band was unwound. When the FW continued to rewind again, this immediately forced the player to bend their knees and begin the eccentric contraction phase of the next repetition. The players were instructed to over-win the kinetic energy with the highest possible mobilization of muscular force at the end of the eccentric movement (~ 80° knee joint angle), and immediately start a new concentric MIVC. During the sets and sessions, the load (Watt) was monitored using the manufacturer’s application (Bluebrain, Kuopio, Finland) on a portable tablet (Samsung Galaxy S4, Samsung Electronics, Daegu, South Korea) connected to the FW device through Bluetooth. The starting inertia at week 2 was set to #1 (0.025 kg·m^− 2^). If the players produced on average > 4 watts·kg^− 1^ from each repetition of one set, the FW size was increased, to #2 (0.05 kg·m^− 2^) and later to “#3” (#1 + #2 = 0.075 kg·m^− 2^) and finally #4 (0.1 kg·m^− 2^).

### Barbell free weight squat group

**Fig. 2:**
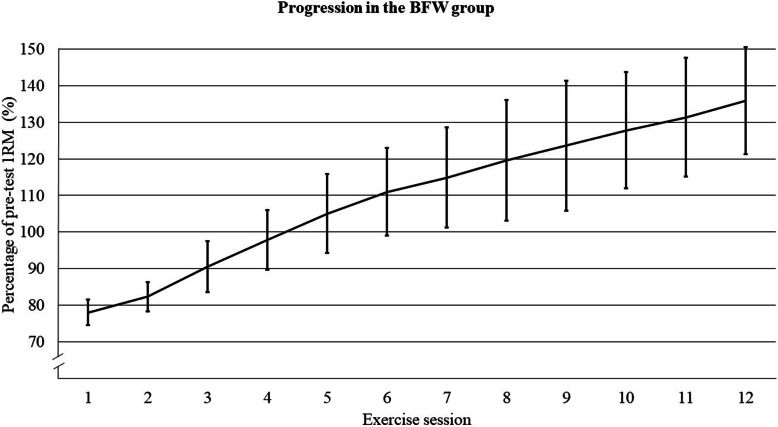
The logged progression of the players in the BFW group. Data are percentage of pre-test 1RM lifted in the final set of the session and shown as mean with error bars as SD. BFW = barbell free weight, 1RM = one repetition maximum, SD = standard deviation

The players in the BFW group performed a specialized warm-up with three sets of progressively increasing intensity in the squat exercise; eight repetitions at 30%-, six repetitions at 50%- and six repetitions at 70% of 1RM, respectively. The first two sessions (week 1) consisted of three sets with eight repetitions at ~ 70% of 1RM. Thereafter, from the third session (week 2), the players were instructed to perform four sets of four repetitions at > 85% of 1RM throughout the remaining sessions with progressively increasing the load with 5 kg if they could perform five repetitions within one set (e.g. if performing 5 repetitions, the load in the next set was increased, which could be set 1, 2, 3 or 4 in the exercise session). Recovery time between sets was set to ≥3 min. Figure 2 illustrates the logged progression of the BFW group.

### Statistical analyses

The Shapiro Wilk test confirmed all data to not deviate from normal distribution, both prior (all *p* ≥ 0.11) and following randomization (all *p* ≥ 0.052), which were confirmed by inspection of the Q-Q plots. We performed paired sample t-tests to assess pre- to post-test changes within groups. One-way univariate analyses of variance (ANOVAs) with Bonferroni corrected post-hoc tests were used to examine differences in baseline characteristics, and in the change score (post-pre) from pre-to post-test between the groups. Effect sizes were calculated as Cohen’s *d* where determination of magnitude was considered according to Rhea’s recommendation for RE interventions of moderately fit individuals; trivial: < 0.35, small: 0.35–0.79, medium: 0.80–1.49, large: ≥1.50 [38]. For pre- to post effect size within groups, we divided the mean change score by the standard deviation (SD) of the change score. We calculated between groups effect size by the pooled SD of the two groups of interest (e.g. FW vs BFW, FW vs control, BFW vs control) divided by the difference in mean change score of the two groups of interest using the following formula:

$$ \sqrt{\frac{\left({n}_1=1\right)\times {SD}_{1^2}+\left({n}_2-1\right)\times {SD}_{2^2}}{n_1+{n}_2-2}/}{m}_1-{m}_2 $$

Where n_1_ and n_2_ represents the groups’ *n*, SD_1_^2^ and SD_2_^2^ represents the groups’ SD squared, m_1_ and m_2_ represents the two groups’ mean change score, respectively. We used Pearson’s correlations to assess the association between the change in sprint time and jump height, respectively, and the change in maximal partial squat strength. We adopted linear regressions to assess whether inclusion of changes in body mass could explain more of the variation in the association than maximal partial squat change alone. We performed a pilot study where we observed a mean decrease of 0.0243 ± (SD) 0.0215 s in the 10-m sprint test following 6 weeks of partial squat exercise at > 85% of 1RM characterized by 4 × 4 repetitions. Sprinting 0.02 m·s^− 1^ faster over 10 m would result in a ~ 10 cm difference, which can be considered a shoulder length ahead of an opponent and thus a game changing and relevant difference [14]. With 80% power and an alpha level of 0.05, we calculated to need 12 participants in each group. We assumed a 25% dropout and thus aimed to recruit at least 45 participants (15 in each group); following dropouts (22.5%), we ended up with 13 (FW), 13 (BFW) and 12 (control) in our three groups for the final analyses. Data are shown as mean ± SD unless otherwise is stated. All statistical analyses were performed using the Statistical Package for Social Sciences (SPSS, Version 26, IBM, Armonk, NY, United States).

## Results

**Fig. 3:**
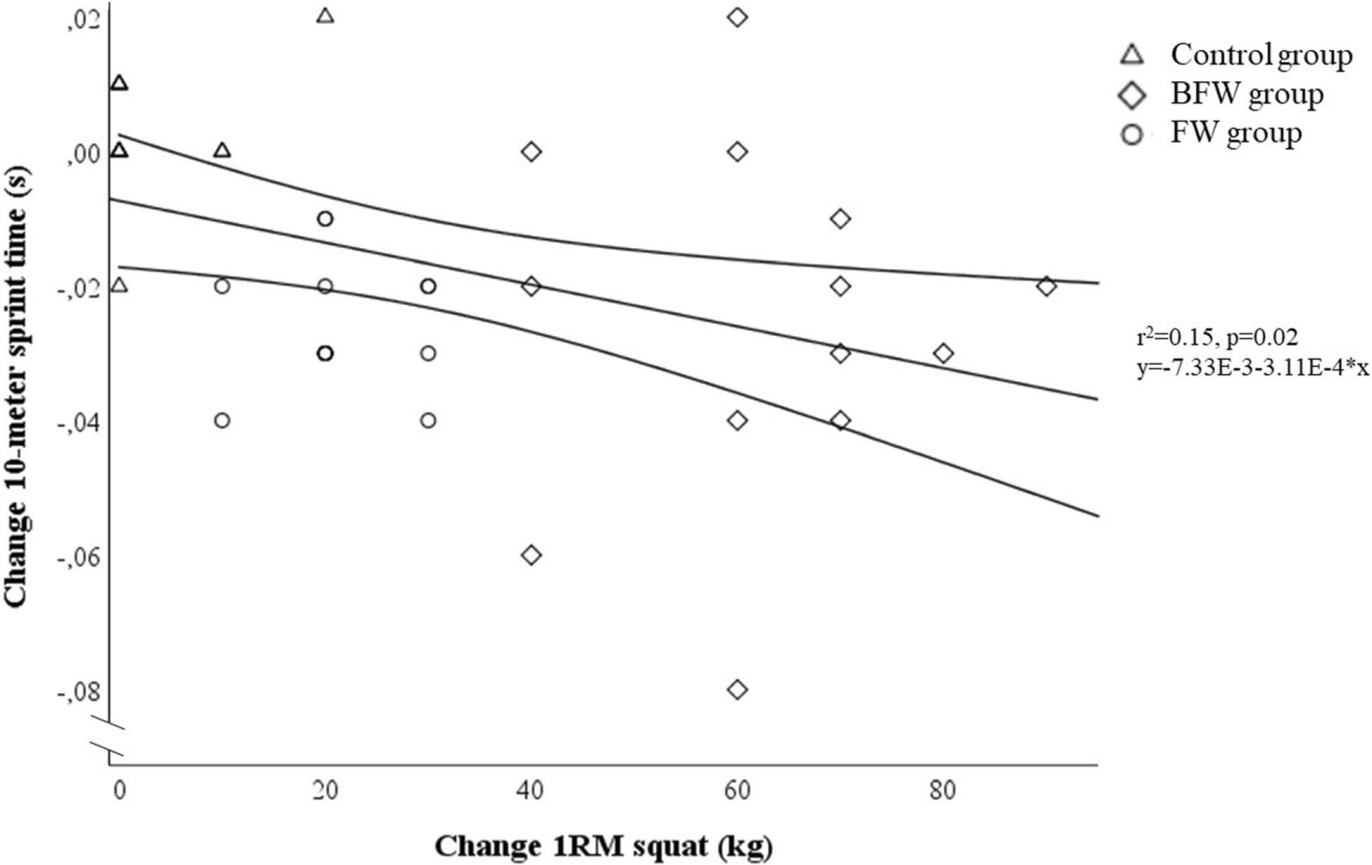
Scatter plot with regression line and 95% confidence intervals of the pre- to post change in 1RM partial squat strength and 10-m sprint time. The bold triangles, squares and circles indicate multiple data points (multiple individuals having the same values). 1RM = one repetition maximum

The pre- and post-test results are presented in Table 3. There were differences in changes in the 10-m sprint test between the groups (between subjects effect: *p* < 0.001), where the FW and the BFW group equally decreased their 10-m sprinting time from pre- to post-test by 2% (between groups: *p* = 1.00, Cohen’s *d:* 0.00*,* pre- to post-test: FW group: *p* < 0.001, Cohen’s *d*: − 0.97; BFW group: *p* = 0.005, Cohen’s *d*: − 0.96), while the control group did not decrease their sprinting time (*p* = 0.39, Cohen’s *d*: 0.26; difference between FW and BFW vs control: both *p* < 0.001, both Cohen’s *d:* − 1.39) (Table 3). The individual change in 10-m sprint time from pre- to post-test and the association with 1RM partial squat change is illustrated in Fig. 3. Two out of the 13 in the FW group did not experience a game changing relevant change in 10-m sprint performance (≥0.02 s decrease in 10-m sprint time; range FW group: 0.02 to − 0.08 s, mean increase: − 0.03 ± 0.01 s). Four out of the 13 in the BFW group (range: − 0.01 to − 0.04 s, mean increase: − 0.03 ± 0.03 s) and 11 out of the 12 players in the control group (0.02 to − 0.02 s, mean increase: 0.003 ± 0.01 s) did not experience a game changing relevant change in 10-m sprint time (Fig. 3).

**Table 3:** Pre- and post-test results

	FW group (***n*** = 13)		BFW group (***n*** = 13)		Control group (***n*** = 12)	
	Pre-test	Post-test	Pre-test	Post-test	Pre-test	Post-test
Body mass (kg)	78.69 ± 7.42	79.33 ± 7.29	78.87 ± 11.98	79.33 ± 12.19	83.13 ± 7.06	83.20 ± 6.68
10 m sprint time (s)	1.75 ± 0.07	1.72 ± 0.07¤*	1.74 ± 0.08	1.71 ± 0.07¤*	1.73 ± 0.04	1.73 ± 0.04
CMJ (cm)	34.38 ± 2.15	37.45 ± 3.46¤*	36.98 ± 3.98	39.75 ± 4.14¤*	35.99 ± 3.79	36.06 ± 3.41
Squat 1RM (kg)	127.69 ± 21.27	149.23 ± 22.16¤*	134.62 ± 26.96	196.92 ± 26.89¤*#	137.50 ± 21.79	140.83 ± 25.39
Squat 1RM scaled (1RM kg·weight kg^-0.67^)	95.27 ± 15.47	111.30 ± 16.01¤*	100.41 ± 19.49	146.94 ± 19.65¤*#	102.23 ± 15.87	104.69 ± 18.50

Data are shown as mean ± SD. ¤significant difference from pre- to post-test, *p* < 0.001, *significant difference in the pre- to post-test change from the control group, *p* < 0.001, #significant difference in the pre- to post-test change from the FW group, *p* < 0.001. *FW* Flywheel, *BFW* Barbell free weight, *CMJ* Countermovement jump, *1RM* One repetition maximum

**Fig. 4:**
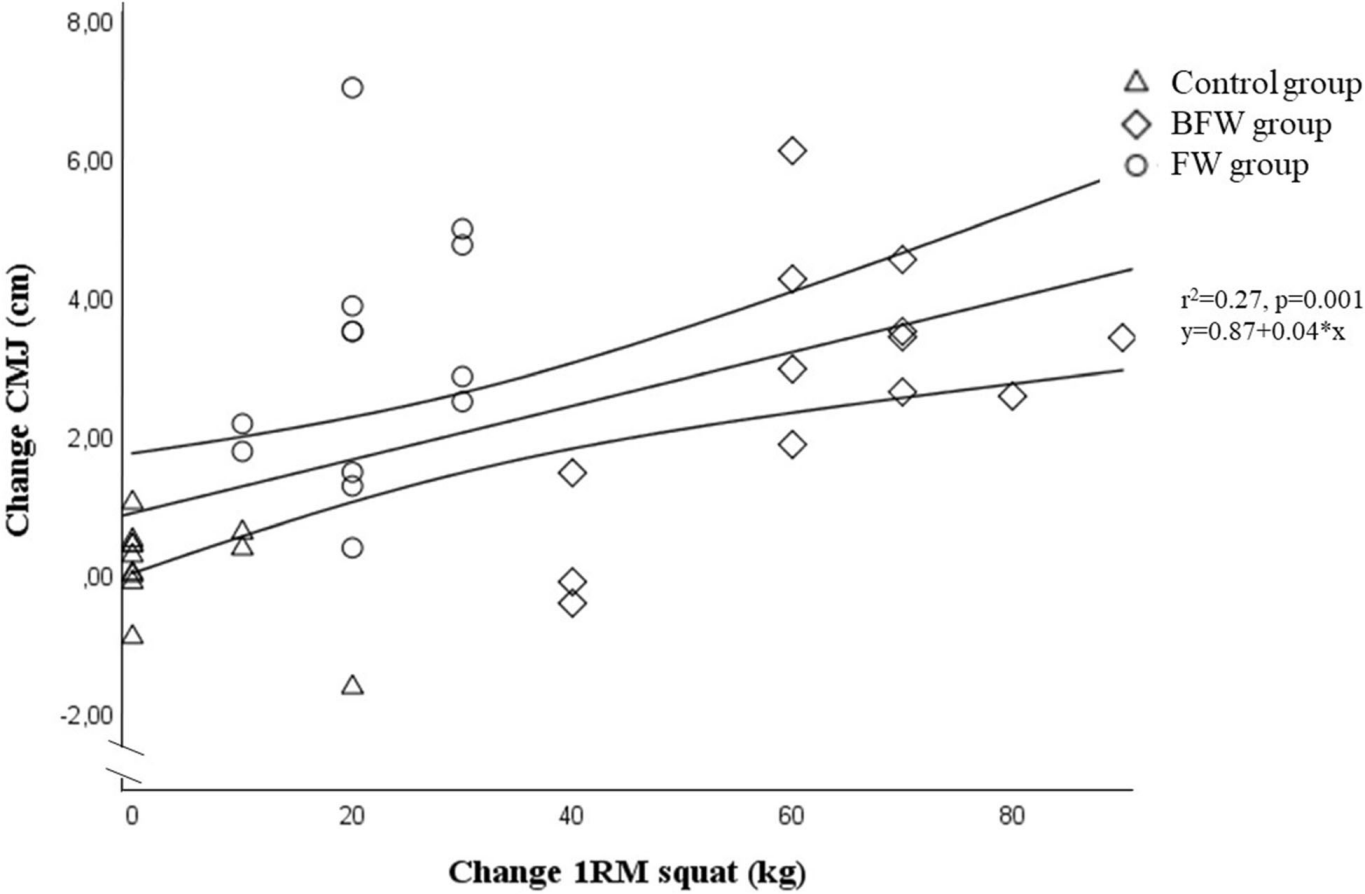
Scatter plot with regression line and 95% confidence intervals of the pre- to post change in 1RM partial squat strength and CMJ. The bold triangles, squares and circles indicate multiple data points (multiple individuals having the same values). 1RM = one repetition maximum, CMJ = countermovement jump

There were differences in changes in the CMJ test between the groups (between subjects effect: *p* < 0.001), where the FW and the BFW group equally increased their jump height in the CMJ test from pre- to post-test by 9 and 8%, respectively (between groups: *p* = 1.00 Cohen’s *d:* − 0.16; pre-to post-test: FW: *p* < 0.001, Cohen’s *d:* 1.70; BFW: *p* < 0.001, Cohen’s *d:* 1.54), while the control group did not increase their jump height (*p* = 0.75, Cohen’s *d:* 0.09; difference between FW and BFW vs control: both *p* < 0.001, Cohen’s *d:* FW vs control: 2.15, BFW vs control: 1.94) (Table 3). The individual CMJ change from pre-to post-test and the association with 1RM partial squat change is illustrated in Fig. 4. All players in FW group experienced an increase in jump height (range 0.37–7.01 cm, mean increase: 3.07 ± 1.80 cm). In the BFW group, 11 out of the 13 increased their jump height (range: − 0.43 to 6.10 cm, mean increase: 2.78 ± 1.80), while seven out of the 12 players in the control group experienced an increased jump height from pre- to post-test (range: − 1.64-1.02 cm, mean increase: 0.07 ± 0.72 cm) (Fig. 4).

There were differences in changes in the 1RM partial squat test between the groups (between subject effect: *p* < 0.001), where the BFW group increased their 1RM squat by 46%, which is more than the FW group’s increase of 17% (difference between groups: *p* < 0.001, Cohen’s *d:* 3.43*,* pre- to post-test: FW: *p* = 0.001, Cohen’s *d:* 3.13, BFW: *p* < 0.001, Cohen’s *d:* 3.17), and the BFW and the FW group increased their 1RM squat more than the control group (difference between FW and BFW vs control: both *p* < 0.001, Cohen´s *d*: FW vs control: 2.71, BFW vs control: 4.93, pre-to post-test control group: *p* = 0.10, Cohen’s *d:* 0.51). When scaling 1RM partial squat strength to the power of 0.67, the results remained unchanged (Table 3). For individual pre- to post 1RM partial squat changes, all players in FW and BFW group increased their 1RM (FW: range: 10–30 kg, mean increase: 21.5 ± 6.9 kg; BFW: 40–90 kg, mean increase: 62.3 ± 15.4), while three out of the 12 players in the control group increased their 1RM (range: 0–20 kg, mean increase: 0.07 ± 0.72 cm) (Figs. 3 and 4).

We observed a negative linear association between the change in maximal partial squat strength and the change in sprint time (1RM: *r* = 0.39, *r*^2^ = 0.15, *p* = 0.02) (Fig. 3). We observed a positive linear association between maximal partial squat strength and jump height (*r* = 0.52, *r*^2^ = 0.27, *p* = 0.001) (Fig. 4). These associations were unchanged when including change in body mass as independent variable, and when changing 1RM to scaled 1RM (data not shown).

## Discussion

In this randomized controlled trial of recreationally active football players, FW and BFW HL squats equally improved 10-m sprinting time and CMJ height while BFW HL squats was superior to FW squats in improving maximal partial squat strength. Finally, we observed linear associations between changes in maximal partial squat strength and changes in 10-m sprinting time and CMJ, respectively.

The equal improvement for both intervention groups in 10-m sprint time and jump height is in line with the latest systematic review assessing the effect of RE in football players [8], and also with previous studies assessing the effect of BFW HL partial squats combined with football sessions [22, 34]. Although not always consistent [39], sprint improvements following FW squats is reported previously [40, 41], while improvements in jump height following FW RE seem to be a consistent observation [39–41].

Although we observed linear associations between improvements in maximal squat strength and improvements in sprint time and jump height, which is in line with the latest review on the effect of RE in football players [8], the explained variances are low (10-m sprint change: 15% (*r*^2^ = 0.15), Fig. 3; CMJ change: 27% (*r*^2^ = 0.27), Fig. 4), indicating that other factors than increased maximal squat strength may explain the improved 10-m sprint and jump performance. These similar improvements between the BFW and FW groups are likely explained by neuromuscular adaptations induced by MIVCs [12]. For example, using novel high density surface electromyography recordings, a recent study showed an increased motor unit discharge rate accompanied by a decreased motor unit recruitment threshold following 4 weeks of isometric MIVCs [42]. Moreover, it seems that peak rate of force development is associated with peak motor unit discharge rate, which also seem to be generated prior to maximal force development [16], which thus seem to explain the underlying neural mechanisms for improvements of high velocity movements following RE [12].

However, it is reported that neural adaptations preliminary occurs within the first 1–2 weeks of RE [25, 43]. Thus, although the strength of the associations between change in sprint time or jump height and change in maximal squat strength were unchanged when including body mass change as independent variable, we cannot rule out whether our 6 week long intervention induced morphological changes (e.g. increase in pennation angle, fascicle length and cross-sectional area), which normally occur as a result of longer exercise programs. For example, a previous study assessing the effect of FW RE revealed changes in muscle fascicle length and pennation angle, which was paralleled with hypertrophy gains [44].

The BFW group experienced a more than two-fold larger increase in 1RM squat (46%) than the FW group (17%). The 17% increase in the FW group is in line with previous reported increases following squat RE in football players [8], while the 46% increase in the BFW group is towards the highest reported increases in 1RM partial squat in the literature for football players (52%) [8, 34]. A meta-analysis reported that FW RE is not superior to traditional RE for strength improvements [45], which corroborate our findings. Nevertheless, the difference in 1RM squat strength between the BFW and the FW group in our study is likely an effect of test specificity where the exercise performed by the BFW group was isotonic to the test; this is shown previously for the squat exercise [46]. Consequently, we urge for cautious interpretation when comparing 1RM gains between the BFW and FW group.

A previous meta-analysis comparing concentric and eccentric RE reported superior gains in maximal strength following eccentric RE [24]. However, their stratified analysis of exercise intensity revealed no differences between the two exercise modalities [24]. In fact, in studies comparing solely concentric low intensity (75% of 1RM) contractions with concentric and subsequent eccentric overload contractions (> 100% of 1RM), superior 1RM gains are reported from subsequent eccentric overload [47, 48]. While studies comparing solely concentric higher intensity (maximal 6- and 10RM and > 85% of 1RM) with subsequent eccentric overload reported similar gains in 1RM [49, 50]. This may suggest that as long as the concentric phase is performed with heavy loads (~ ≥ 85% of 1RM), no extra maximal strength gains can be derived from additional eccentric overload [24]. This indicate that high external loads (> 85% of 1RM) should be applied to easily recruit the higher threshold motor units [14], which is responsible for the highest force productions [13].

### Strengths

To our knowledge, this is the first randomized controlled trial comparing FW RE to HL RE practices for maintaining [21] and improving [22, 23] sprint and jump height performance in football. Due to the comparison in our study, one can assess the applicability of FW RE in football. Such information is highly applicable for coaches in football clubs, which should use the best practice in relation to total exercise load to optimize performance of the players, at least in elite clubs.

### Limitations

Some limitations need to be addressed. First, football involves multiple changes of direction at high velocities [51]. As changes of direction involves decelerations and subsequently accelerations in a different direction, the ability to utilize the elastic energy stored in tissues from deceleration during eccentric contractions into a subsequent concentric acceleration phase can be decisive in football [51]. Flywheel RE comprise of such decelerations with high force production, and FW RE is found to improve changes of directions [52]. We did not assess the ability to perform changes of direction our study. Future research investigating whether FW or HL BFW squat exercise results in superior performance in changes of direction tests is warranted.

Further, we did not match exercise intensity between the two intervention groups, which introduce the possibility of the external loads employed in the interventions influencing our results (i.e. the exercise intensity per se, not exercise modality). One study demonstrated that increasing FW inertia increases coupling time (transition from eccentric to concentric contraction during the movement) and reduces power output [53]. Thus, increasing FW inertia might have hindered maximal improvements in high velocity movements (sprint and jump height) for the players in the FW group. However, force production increased by increasing inertia [53] and the intended velocity per se (not actual movement velocity) is responsible for improving high velocity movements following RE [12]. As increasing force production with increasing inertia can be considered higher load RE than not increasing inertia, we increased inertia following mean > 4 watts∙kg^− 1^ in one set to label both intervention groups’ exercise intensity “HL RE” and make exercise intensity between groups more comparable. Thus, we tried to keep similar progression in exercise load in both intervention groups, where reaching a certain limit (FW: > 4 watts∙kg^− 1^, BFW: ≥ 5 repetitions) resulted in an increased load in the next set. This also ensured individualized progression, as highlighted as an important factor for optimizing improvements in sprint performance from FW RE [53].

Furthermore, by performing 4 × 4 repetitions and increasing load when reaching five repetitions in the BFW, without any mid-test 1RM to adjust relative load, there could have been a possibility of some players in the BFW group exercising at < 85% of their actual 1RM as their actual 1RM increases during the intervention. However, this protocol is proved highly effective in improving maximal strength [21, 22, 34] and moreover, the increase from week to week was high in this group (Fig. 2), ultimately leading to a 46% increase in 1RM, which is towards the highest reported increases in 1RM in football players [8].

Hamstring muscle strength is associated with sprint performance [54–56], and antagonist co-contraction may have contributed to the increase in force production by an exercise-induced increase in reciprocal inhibition [57]. As both intervention groups performed the Nordic Hamstring exercise, the control group should also have performed this exercise allowing us to solely compare the effects of FW and BFW squats. However, the potential effects of Nordic Hamstring on sprint and jump height performance in our two intervention groups should influence our results in similar proportional order. Nevertheless, it seems that antagonist co-contraction plays a greater role in joint protection in RE, suggesting that they may play a minor role in the actual movement velocity [57]. Moreover, the effect on sprint performance following Nordic hamstring exercise is usually small [58, 59] or non-existing [60].

Finally, this study included recreationally active football players. Elite football players are reported to sprint faster than lower level players [1] and have a larger total exercise load resulting in limited recovery time between exercise sessions [20]. Whether differences in sprints, jump height and maximal strength gains from FW and BFW squats would be present in elite football players are currently unknown. However, as the players in our study experienced similar gains from BFW squats on sprint, jump height and 1RM partial squat as previously reported in elite football players [8, 22, 34], one may consider our study’s findings generalizable to elite football players, at least until proven otherwise by future research.

## Conclusion

Squats carried out with FWs or HL BFWs where both are performed with MIVCs and combined with football sessions, were equally effective in improving sprint time and jump height in football players. The BFW group experienced a more than two-fold larger increase in maximal partial squat strength than the FW group. This presents FW RE as an alternative to HL free weight RE for improving high velocity movements in football players.

## Supplementary information

**Additional file 1.** Supplementary file: zip-file containing the dataset analyzed for this study.

## Data Availability

All data generated or analysed during this study are included in this published article and its supplementary information files.

## Abbreviations

RE: Resistance exercise
FW: Flywheel
HL: High load
BFW: Barbell free weight
SD: Standard deviation
1RM: One repetition maximum
MIVC: Maximal intended velocity contraction
